# Evaluation of Uterine Artery Doppler and Estrogen Milieu in Oocyte Donation Pregnancies—A Pilot Study

**DOI:** 10.3390/diagnostics10050254

**Published:** 2020-04-26

**Authors:** Luca Mandia, Paolo Cavoretto, Piergiorgio Duca, Massimo Candiani, Irene Cetin, Valeria Savasi

**Affiliations:** 1 Unit of Obstetrics and Gynecology, Department of Biomedical and Clinical Sciences, ASST Fatebenefratelli Sacco, University of Milan, Via G. B. Grassi, 74, 20157 Milan, Italy; 2 Centre for Fetal Research Giorgio Pardi, University of Milan, 20100 Milan, Italy; 3 Department of Obstetrics and Gynecology, IRCCS Ospedale San Raffaele, University Vita-Salute San Raffaele, 20132 Milan, Italy; 4 Statistical and Biometry Unit, Department of Biomedical and Clinical Sciences, University of Milan, Via G. B. Grassi, 74, 20157 Milan, Italy

**Keywords:** IVF, oocyte donation, uterine arteries Doppler, estrogen milieu, first trimester

## Abstract

Oocyte donations (OD) represent 4.5% of all in vitro fertilization (IVF) cycles. While OD pregnancies face increased risks of obstetrical complications, especially pregnancy-induced hypertension and pre-eclampsia (PE), little is known about the physiology and the physiopathology of placentation. We performed a prospective case-control study to analyze uterine artery Doppler pulsatility index (UtA-PI) and serum maternal 17β-estradiol (17β-E) at 11 + 0 to 13 + 6 weeks’ gestation in singleton pregnancies with different modes of conception. Study groups were: 55 OD, 48 IVF with autologous oocytes from fresh cycles (Autologous-Fresh IVF), 10 IVF with autologous oocytes from frozen cycles (Autologous-Frozen IVF) and 122 spontaneously conceived pregnancies (SC). The mean UtA-PI and serum maternal 17β-E at 11 to 13 + 6 weeks were significantly lower in OD as compared to SC and autologous IVF, either from fresh or frozen cycles. Oocyte donation presents lower UtA-PI and lower serum 17β-E in the first trimester of pregnancy. The etiology of these particularr differences is likely multifactorial and deserves further investigation.

## 1. Introduction

The number of oocyte donation (OD) cycles has dramatically increased in recent years, and now represent 4.5% of all in vitro fertilization (IVF) [1,2]. Independent studies have shown that OD pregnancies face increased risks of obstetrical complications, especially pre-eclampsia (PE), as compared to spontaneously conceived pregnancies (SC) and to IVF with homologous oocytes [3,4]. While these clinical aspects are considered to be a consequence of poor placentation or abnormal cardiovascular adaptation to pregnancy, little is known about the physiology and the physiopathology of the interaction between the embryo and the mother. We know that estrogens have an essential role in placentation, promoting angiogenesis and development of placental villous vascularity [5]. Recently it was demonstrated that the hyper-estrogenic milieu generated during ovarian stimulation may create a suboptimal environment that damages implantation, which leads to placental dysfunction and low birth weight [6].

Our group has demonstrated that OD pregnancies present a lower uterine arteries pulsatility index (UtA-PI) at 11 + 0 to 13 + 6 weeks as compared to both homologous IVF and SC [7]. Uterine artery Doppler is a non-invasive method that can detect abnormal placentation and can contribute to predictions of preterm pre-eclampsia [8,9]. In this prospective study, we tested the hypothesis that in OD pregnancies, uterine impedance to flow measured with UtA-PI could be different due to the estrogen milieu during early pregnancy and we analyzed maternal 17 β-estradiol serum concentration (17β-E) in SC, autologous IVF (fresh or frozen embryos) and OD pregnancies.

## 2. Materials and Methods

### 2.1. Population

We performed a prospective case-control pilot study to analyze UtA-PI and 17β-E at 11 + 0 to 13 + 6 weeks’ gestation. Four groups of pregnancies were studied: OD, IVF with autologous oocytes from fresh cycles (Autologous-Fresh IVF), IVF with autologous oocytes from frozen cycles (Autologous-Frozen IVF) and SC. Inclusion criteria were: singleton pregnancies from nulliparous patients with different identified methods of assisted conception or normal pregnancies with SC. Patients attending our clinic for routine prenatal risk assessment for chromosomal abnormalities in the first trimester were enrolled in the study between January 2014 and January 2016. Exclusion criteria were (1) pre-existing maternal diseases (diabetes, chronic hypertension, renal disease, other significant diseases); (2) fetal structural or chromosomal anomalies; (3) vaginal bleeding or threatened miscarriage; (4) multiple pregnancies or pregnancies with vanishing twins above 6 weeks of development (crown rump length > 10 mm); and (5) smoking status. All women underwent OD for low ovarian reserve and the ICSI fertilization technique was used in the reproductive laboratory for all patients. Data from patients that were lost to follow-up were excluded from final analysis. The recorded patient characteristics included maternal age, racial origin (Caucasian, Afro-Caribbean, South Asian, East Asian or mixed), method of conception, personal medical and obstetric history (previous pregnancies before 24 weeks). Maternal weight and height were measured and the body mass index was calculated as kg/m^2^.

**Figure 1:**
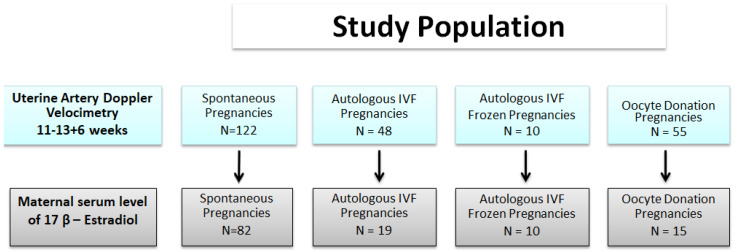
The study population.

The OD study group included 55 OD IVF/ICSI (oocyte recipients) pregnancies compared to 48 pregnancies obtained with autologous IVF/ICSI from fresh cycles, 10 pregnancies obtained with autologous IVF/ICSI from frozen cycles and 122 singleton SC pregnancies as described in Figure 1. Autologous-Frozen IVF pregnancies involved the transfer of thawed blastocysts that had been cryopreserved by vitrification. In our center, the protocol for frozen blastocyst transfer is based on hormone replacement therapy for all women, whereas autologous IVF were all treated with identical standard protocol. In all OD and autologous frozen IVF/ICSI pregnancies, the women were taking Estradiol Valerate (three tablets of 2 mg per day from endometrial preparation until the end of first trimester) and post-transfer Natural Progesterone 600 mg vaginally per day until the end of first trimester. Outcomes of pregnancy were recorded with special reference to gestational hypertension, pre-eclampsia or fetal growth restriction. Written informed consent was obtained from all the women who agreed to participate in the study, which was approved by the Ethics Committee of the Hospital (Ethics approval number N.48 2013).

### 2.2. Methodology

Ultrasound and Doppler examinations were performed by two sonographers (L.M. and P.C.) who have certification of competence in 11 + 0 to 13 + 6 week scanning and Doppler ultrasound from The Fetal Medicine Foundation (https://fetalmedicine.org/). All ultrasound examinations were performed using Voluson E8/E10 or Voluson 730 Expert ultrasound machines equipped with convex multi-frequency transducers (GE Medical Systems, Milwaukee, WI, USA). Transabdominal ultrasound examination was carried out for evaluation of fetal anatomy, measurements of fetal crown–rump length, nuchal translucency thickness and UtA-PI, which was calculated by the following equation: UtA-PI = (peak systolic velocity–end diastolic velocity)/time averaged velocity. Uterine Doppler studies were performed transabdominally, a sagittal section of the uterus was obtained, the cervical canal and internal cervical os were identified and then the transducer was tilted slightly to the left and to the right. Color Doppler was then applied to visualize the paracervical loops of each uterine artery. Pulsed wave Doppler was used with the sampling gate set at 2 mm to cover the whole vessel. Care was taken to ensure that the angle of insonation was less than 30 before applying spectral Doppler interrogation. When three similar consecutive waveforms were obtained, left and right UtA-PI was measured by manual tracing of the waves, then, the mean left and right UtA-PI was calculated and used for analysis.

Maternal venous concentrations of 17β-E were analyzed in 82 SC, 19 Autologous-Fresh IVF, 10 Autologous-Frozen IVF and 15 OD pregnancies, which were selected randomly after the ultrasound examination at 11 + 0 to 13 + 6 weeks’ gestation. Serum samples were processed and assayed for 17β-E using the Electro Chemo Luminescence in Immunoassay (ECLIA–Elecsys/Cobas^®^) method in the same laboratory. The choice to select a smaller cohort of patients in which to analyze maternal serum 17β-E was due to limits imposed by the cost of the study. For spontaneous pregnancies we performed a more detailed analysis due to the greater size of the cohort. We correlated the maternal serum level of 17β-E and mean UtA-PI at 11–13 + 6 weeks. In order to explore the possible influence of fetal gender on 17β-E maternal serum concentration, we analyzed 66 spontaneous pregnancies with the fetal gender registered at birth, 22 males and 42 females.

### 2.3. Statistical Analysis

Statistical analysis was performed using the Statistical Package for Social Science version 17.0 (SPSS Inc., Chicago, IL, USA). The usual descriptive statistics were computed considering four main groups: (1) spontaneous pregnancies, (2) autologous IVF pregnancies from fresh cycles (Autologous IVF), (3) autologous IVF pregnancies from frozen cycles (Autologous-Frozen IVF), and (4) oocyte donation pregnancies (OD). The significance of the differences between groups’ means was tested using one-way analysis of variance (ANOVA) and following the Fisher’s Least Square Difference method. Means, standard deviations, medians and quartiles, when appropriate, were used for description. The non-parametric Kruskal-Wallis or Mann-Whitney U test was used to test differences in median levels of maternal serum 17β-E. *p* values were reported, and a *p* less than 0.05 (two tails) was considered as statistically significant. Finally, 95% confidence intervals were also computed. To test the difference in estrogen concentration in relation to fetal gender, a Student’s t-Test was performed on log-transformed maternal serum estrogen concentrations.

## 3. Results

This study presents the results of UtA-PI and 17β-E measurements obtained at 11 to 14 weeks gestation for 235 nulliparous, non-smoking women with different methods of conception. Table 1 presents the baseline characteristics of the maternal and fetal study population. The mean maternal age is significantly different among OD and other groups, as oocyte recipients were significantly older than IVF and spontaneous pregnancies.

**Figure 2:**
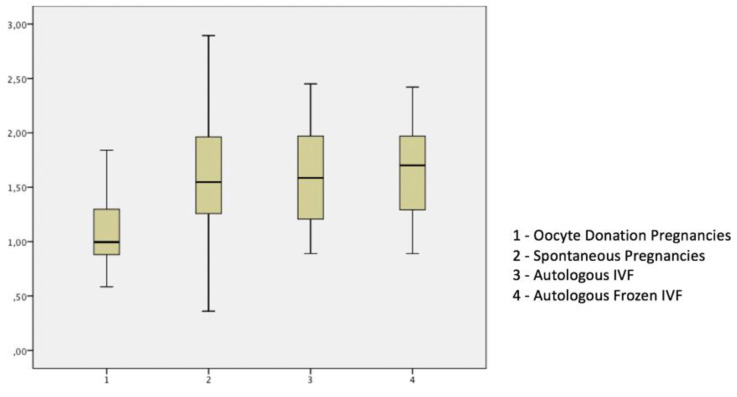
Box and whisker plot of uterine arteries pulsatility index at 11 to 13 + 6 weeks in study groups.

Table 2 and Figure 2 present the results of the UtA-PI at 11 to 13 + 6 weeks. The mean UtA-PI was significantly lower in OD compared to SC, autologous-fresh IVF and autologous-frozen IVF pregnancies. UtA-PI was inversely and significantly correlated to gestational age, with a similar trend in all groups (*p* < 0.01).

**Figure 3:**
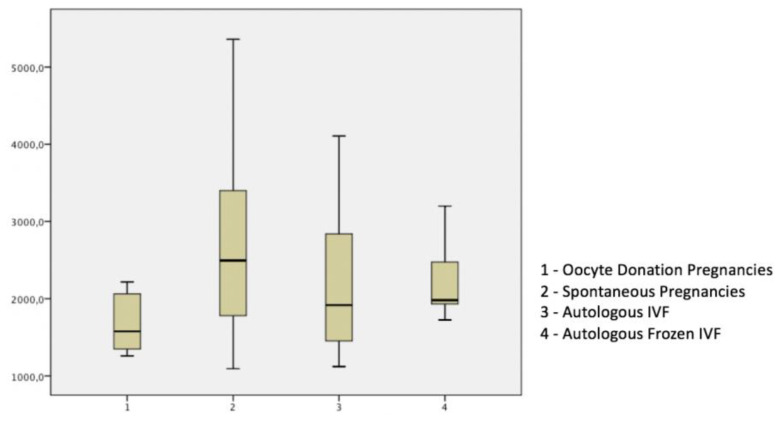
Maternal serum level of 17 β–estradiol (pg/mL) at 11 to 13 + 6 weeks.

Table 2 and Figure 3 show that maternal serum levels of 17β–E in OD (oocyte recipients) were significantly lower as compared to each IVF group and SC pregnancies.

No correlation was demonstrated between maternal serum levels of 17β-E and mean UtA-PI (*p* > 0.05). In SC pregnancies, due to the size of the study group, we searched for a possible role of fetal gender on maternal 17β-E levels. We found a higher concentration of 17β-E in female fetuses with a mean value of 3204 pg/mL (DS 1634) compared to the mean value of male fetuses of 2436 pg/mL (DS 1170). After logarithm transformation, the Student’s t-Test demonstrated a statistically significant higher maternal serum level of 17β-E value in female fetuses (*p* value = 0.0222, 95% IC 0.01742–0.21855), with a female/male ratio of 17β-E maternal serum concentration of 1.31 (IC 95%: 1.04–1.65). The outcomes of pregnancy were recorded: gestational hypertension occurred in 2 OD and 2 SC cases and none of the other study groups (*p* > 0.05); pre-eclampsia occurred in 2 OD and 2 SC cases and none of the other study groups (*p* > 0.05); and fetal growth restriction occurred in 1 cases of SC and none of the other study groups (*p* > 0.05).

## 4. Discussion

### 4.1. Summary of Main Findings

This prospective study confirms previous conclusions made by our group regarding significantly lower UtA-PI in OD pregnancies at 11–14 weeks, as compared to both autologous IVF and spontaneous pregnancies [7]. Additionally, the study shows lower maternal serum concentration of 17β-E in OD pregnancy at 11–14 weeks as compared to both IVFs (with fresh or frozen embryo transfer) and SC pregnancies, without significant correlation to Doppler findings. Finally, in spontaneously conceived pregnancies, 17β-E was higher when the fetus was female.

### 4.2. Interpretation

Ultrasound examinations have a major role as an intraoperative aid for IVF procedures [10,11] and also in the prenatal risk assessment of the resulting pregnancies. This is principally due to uterine artery Doppler studies [7,12,13].

Research work by another group on the same gestational period failed to show significant differences in uterine artery Doppler velocimetry of 109 oocyte donation pregnancies compared to IVF or spontaneous pregnancies [14]. Since UtA-PI is dependent of gestational age, the authors converted the PI value into multiples of the expected median (MoMs), which are calculated from Fetal Medicine Foundation references for singleton pregnancies, after correction for gestational age, body mass index and maternal ethnicity. It is possible that this represents an analytical bias: in fact, MoMs are derived from the ratio of the value obtained on the expected median. However, the correct expected median value for oocyte donation remains unknown, as no reference curve is available; therefore, the use of the expected median of spontaneous pregnancies here is questionable.

To our knowledge, only one previous study has described the influences of estrogens concentrations on human uterine arteries Doppler parameters [15]. This research was performed on 44 women with spontaneous singleton pregnancies between 5 and 16 weeks and found an inverse correlation between 17β-E (gradually rising, in the study period) and the progressive fall of the uterine resistance as measured by the RI (*p* < 0.001). This suggested that 17β-E may play a role in the modifications of the uterine arterial system [13]. There are no data on maternal serum concentrations of 17β–E in OD at 11 to 13 + 6 weeks of pregnancies.

Recent evidence shows that homologous IVF/ICSI pregnancies with frozen blastocyst transfer as compared to fresh blastocyst transfer present lower UtA-PI from 7 to 37 weeks, greater estrogen peak at conception and greater fetal growth [14]. Since most OD are performed using ovocyte or embryonic cryopreservation techniques, such knowledge may be relevant for further studies on this topic. However, it is likely that Doppler and the hormonal alterations described may have a multifactorial etiology, whereby intrinsic differences in the patient’s characteristics (e.g., cardiovascular and metabolic function) as well as procedure-related factors may contribute to different extent, favoring or hampering placental and fetal development. In addition, recent work suggests that cardiovascular function is impaired and pre-eclampsia risk is elevated in women conceiving by IVF in the absence of a corpus luteum, which indicates the importance of the hormonal milieu in the early stages of pregnancy [16].

The observed biophysical and biochemical differences could reflect alterations in the first stages of pregnancy due to oocyte donation, maternal ageing, cardiovascular adaptation and other variables. The fertilization technique by itself is probably not the cause of this difference, as in our study the reproductive laboratory used ICSI for all patients. Unfortunately, we cannot exclude the influence of maternal age in our results. In fact, maternal ageing and associated low ovarian reserve characterize most OD patients, making it very difficult to match groups of OD and autologous IVF for this variable.

Our group recently reported differences in placental free β-hCG levels and both free β-hCG and PAPP-A levels at 11 to 13 + 6 weeks, both in autologous IVF/ICSI and OD pregnancies [17,18]. Despite the critical importance of endocrine characteristics of IVF/ICSI pregnancies in relation to pregnancy outcome and placental function, there is a paucity of data that evaluates additional hormones.

In animal models, reduction in uterine vascular resistance may result from fluctuations in circulating steroids, proteins with the essential role of estrogens in promoting placental villous blood vessels [5,19]. In pregnant women, no differences were found in the mean RI of uterine and spiral arteries between intrauterine and ectopic human pregnancies, suggesting that uterine vascular changes occur even if placentation occurs outside the uterine cavity [20]. In baboon pregnancy, it was demonstrated that prematurely elevating estrogen levels in the first trimester can suppress trophoblastic remodeling of the uterine spiral arteries [21]. This evidence needs to be considered in OD cycles. In fact, in OD pregnancies, hormonal administration for endometrial preparation may play a pivotal role in supporting the paracrine and autocrine inputs that drive decidualization, implantation and placentation [22].

Our finding of lower maternal serum concentration of 17β-E in OD pregnancies at 11 to 13 + 6 weeks is surprising. In fact, we expected higher concentrations of 17β-E in OD pregnancies as compared to SC or autologous IVF pregnancies as a result of the addition of doses administered orally compared to those produced by the placenta. We can speculate that this result could be the consequence of at least two possibilities: first, OD placentas produce less estrogen, probably due to their smaller volumes [6]; and second, oral estrogen administration inhibits placental production, in a sort of feedback mechanism that is common in many endocrine systems.

The present study has some limitations. First, the sample size of the study groups was relatively small. This is partly due to difficult referrals of many uncomplicated IVF/ICSI to high-risk pregnancies unit and partly due to the reticence of many patients with OD to disclose the method of conception in order to maintain their privacy. Second, we could not record data regarding the histological examination of the placenta as a significant proportion of the deliveries did not take place in our centers. Third, it was not possible to collect the complete history of donors and stratify patients according to the cause of infertility, particularly endometriosis, which has an important association with abnormal outcomes of IVF, with or without OD [23,24]. Fourth, it was impossible in this explorative preliminary study to evaluate maternal serum concentrations of other biological forms of estrogen and progestogen. The choice to analyze 17β-estradiol in OD was based on the pharmacokinetic and pharmacodynamics profile of estradiol valerate [25,26]. This study has several strengths. First, to our knowledge there are no published papers on this topic, therefore it provides novel evidence of the metabolism and concentration of estrogens in OD pregnancies. Second, this knowledge is consistent with our recent findings of lower UtA-PI in OD pregnancy [7]. Third, the research presents clear and homogeneous study groups with very narrow gestational age and similar characteristics. Fourth, it shows clearly that 17β-E and UtA-PI are not correlated and therefore, if these results are confirmed, they could be tested individually in future studies. Fifth, if these results are independently confirmed, this knowledge may facilitate further research on the optimization of estrogen levels in OD that aims to improve pregnancy outcomes. 

## 5. Conclusions

Our study shows that OD pregnancies present lower UtA-PI and 17β–E levels as compared to homologous IVFs and SC. These data may have important implications for clinical practice. First, considering that first trimester evaluation of UtA-PI is the backbone for screening for PE, it may be necessary to build reference ranges specifically for OD pregnancies and adjust measurements for this mode of conception, in order to improve the test performance. Second, since estrogen and progesterone normality curves are available for spontaneous pregnancy [27], it will be possible to compare these to those observed in OD. Future larger prospective studies are required to clarify the physiologic ranges of the maternal endocrine profile. This knowledge could lead to the development of hormonal replacement therapies that are capable of balancing hormonal levels of OD pregnancies closer to that of normal first trimester biology and perhaps improving the pregnancy outcome.

## Figures and Tables

**Table 1:** Maternal characteristics of the study population at 11 to 13 + 6 weeks.

	Spontaneous Pregnancies *n* = 122	Autologous IVF Pregnancies *n* = 48	Autologous Frozen IVF Pregnancies *n* = 10	OD * Pregnancies *n* = 55	
	Media (DS)	Media (DS)	Media (DS)	Media (DS)	*p* **
Age	32.8 (5.02)	36.6 (4.24)	34.4 (4.87)	43. 2 (4.74)	*p* < 0.05 ^a,b,c^
BMI	22.4 (3.78)	23.0 (3.86)	22.3 (3.76)	22.4 (3,61)	ns
GA	12.4 (0.53)	12.3 (0.68)	12.5 (0.53)	12.2 (0.60)	ns
CRL	61.3 (6.86)	61.5 (9.17)	62.4 (7.06)	59.7 (7.97)	Ns

GA: Gestational Age; CRL: Crown Rump Length; IVF: In Vitro Fertilization; OD: Oocyte Donation Pregnancies; * Oocyte recipients; ^a^: OD vs. Spontaneous conceived pregnancies; ^b^: OD vs. Autologous IVF; ^c^: OD vs. Autologous Frozen IVF; ** Mann-Whitney U test.

**Table 2:** Uterine arteries pulsatility index and maternal serum level of 17 β–estradiol (pg/mL).

	Spontaneous Pregnancies	Autologous IVF Pregnancies	Autologous Frozen IVF Pregnancies	OD Pregnancies *	
	Mean (SD)	Mean (SD)	Mean (SD)	Mean (SD)	*p* **
Uta PI 11–13 + 6 weeks	1.679 (0.456)	1.706 (0.481)	1.692 (0.466)	1.415 (0.486)	*p* < 0.05 ^a,b,c^
17 β-Estradiol pg/ml	2827.22 (1495.9)	2532.4.50 (1564.38)	2285.36 (592.78)	1674.23 (372.1)	*p* < 0.05 ^a,b,c^

IVF, in vitro fertilization; OD, Oocyte Donation; * Oocyte recipients; ^a^: OD vs. Spontaneous Pregnancies; ^b^: OD vs. Autologous IVF; ^c^: OD vs. Autologous Frozen IVF; ** ANOVA.
